# The Importance of Autonomous Regulation for Students' Successful Translation of Intentions into Behavior Change via Planning

**DOI:** 10.4061/2011/697856

**Published:** 2011-08-07

**Authors:** Dian Sheng Cao, Sonia Lippke, Wei Liu

**Affiliations:** ^1^Department of Psychology and Education, Yangzhou University, Yangzhou 225009, China; ^2^Jacobs Center on Lifelong Learning and Institutional Development, Jacobs University Bremen, Campus Ring 1 (Research V), 28759 Bremen, Germany; ^3^Institute of Applied Psychology, Jiangsu University, Zherjiang 212007, China

## Abstract

Physical activity has a high prevention potential in adolescents. This study investigated the relations between physical activity and intention, autonomous regulation, and planning. We hypothesized that planning mediates the relationship between intention and behavior and that this mediation should depend on the level of autonomous regulation. Stratified randomization sampling method was administered to assemble a sample of *N* = 534 students among two schools in China. To test the hypothesis, autonomous regulation, intention, and physical activity were assessed at baseline as well as planning and follow-up physical activity four weeks after the pretest. A moderated mediation model confirmed that planning mediated the intention-behavior relation with the effect of planning being moderated by autonomous regulation. Study results demonstrated that autonomous regulation facilitated the translation of intention into behavior change via planning. To promote physical activity among adolescents, interventions targeting planning and autonomous regulation might facilitate successful translation of intentions into behavior change.

## 1. Introduction

There are lots of benefits from physical activity (PA) engagement. Regular physical activity participation can prevent premature mortality, coronary heart disease, as well as the prevalence of overweight and obesity and reduce the risk of diabetes 2, cardiovascular disease, and some types of cancer in adulthood [1–3]. Regular physical activity participation can also benefit psychological health by reducing depression and anxiety and increasing self-esteem and life satisfaction [4, 5]. During childhood and adolescence it had short-term as well as long-term effects on health [6]. Some studies revealed that formation of exercise habits during adolescence is an important foundation for physical activity in older age [7, 8]. 

Even with all those benefits from physical activity, lots of previous studies showed that adolescence undergoes a steep physical activity decline. At present the prevalence of physical inactivity is decreasing, not just in western developed countries [9] but also in developing countries such as in China [10]. Study results from Sun et al. showed that only about one third of the adolescents accomplished the recommended daily rate of about one hour of regular participation. Thus, it is important to have a better understanding of those factors that affect students to be physically active.

In preventive medicine, intention was comprehensively used as a predictor of behavior change. Different theories, such as protection motivation theory [11], theory of planned behavior [12], and health action process approach [13], include intention as a main component of predicting behavior change. However, even with the comprehensive use of intention in the prediction of behavior change, high intention did not guarantee subsequent actual behavior change. Studies showed that there existed a gap between intention and behavior [14]. 

In order to bridge the intention-behavior gap, some self-regulatory variables should be additionally regarded. Evidence shows that volitional factors—such as planning (implementation intention) specifying when, where and how to carry out the intention—are effective in initiation and maintenance of the intended behavior [15]. Even though planning was found to be a strong predictor of behavior, it can be expected that not everyone benefits to the same extent from the same planning intervention program. Planning's mediating effect might depend on some other influential variables. Koestner et al. showed that the kind of motivation, that is, whether individuals are autonomously regulated, interacts with the effectiveness of planning [16, 17]. Autonomous regulation is characterized by goals that reflect personal interests and values. In contrast, goals which reflect a feeling of being controlled by external pressures characterize rather nonautonomous regulation [6].

Autonomously regulated behaviors are those performed for the satisfaction perceived by engaging in the activity itself. According to most theories the primary satisfaction associated with autonomously regulated actions are experiences of competence and interest or enjoyment. By contrast, not autonomously regulated behaviors are those that are performed in order to obtain rewards or outcomes that are separate from the behavior itself [18, 19]. In some previous studies high autonomous regulation was a critical factor for exercise adherence whereas low or no autonomous regulation resulted in poor adherence [6]. 

Physical activity participation among adolescents in school environment differed from that of among adults in worksite or clinical context. During school time adolescents can develop a heightened autonomy and start making their own decisions on their behaviors [20]. Students are rather motivated to perform physical activity by enjoyment than by disease prevention. Studies show that enjoyment and autonomous regulation were important for physical activity adoption [6]. Autonomous regulation can also facilitate continued involvement in physical activity in later life [21]. Generally, more autonomous regulation significantly predicts more health behavior [22] and its predictors, such as intention and self-efficacy [6]. However, little is known about the role of autonomous regulation in concert with other predictors of behavior, such as intention and planning. Previous studies have tested different moderators of the intention-planning-behavior relation [23, 24]. The question is whether autonomous regulation can serve as such a moderator of the intention-planning-behavior relation.

We hypothesized that the mediating effect of planning depends on autonomous regulation. Only if autonomous regulation is high, planning helps to translate intentions into behavior. If one feels not in control of the own behavior (low autonomous regulation), then planning does not help him to become more physically active even in face of high intentions. 

## 2. Method

### 2.1. Participants and Procedures

Adolescents from grade 7 to 12 were recruited by stratified randomization sampling method from two high schools in the central region of China. The survey was conducted at two points in time within a 4-week period. 693 adolescents participated in the baseline study and provided valid data on exercise intention, autonomous regulation, and physical activity at pretest. Four weeks later, posttest questionnaires were handed out to those students who completed the baseline study. 534 students completed the follow-up study and provided data on planning and physical activity. The final sample consisted of 534 participants with a mean age of 13.95 years (SD = 1.67) and with 52% girls.

At the two dates of the survey, all the questionnaires (taking about 20 minutes to complete) were handed out by trained study assistants. Informed consent was obtained and the study was performed in accordance with the Helsinki Declaration [25].

### 2.2. Measurement

The questionnaire packets contained assessments of intention, autonomous regulation, planning, behavior and sociodemographic information. All original materials were developed and validated in German and English. The questionnaires were translated into Chinese by a bilingual researcher. 

*Physical activity intention* was assessed with one item worded “I intend to do physical activities for 30 min or longer at least three times per week, or accumulating at least 90 min per week on a regular basis.” Responses could be on a seven-point bipolar Likert score ranging from (−3) completely disagree to (+3) completely agree in this study Cronbach's Alpha was *α* = .83.

*Autonomous regulation* was measured with the behavioral regulation in exercise questionnaire (BREQ) [26] consisting of 16 items. Responses were given on a 7-point Likert score ranging from (1) not at all true, (4) somewhat true, to (7) completely true. The index of autonomous regulation for this scale was computed using the following equation to combine the subscale scores (Motivation Index = 2 × Intrinsic + Identified − Introjected − 2 × External [26]). Negative numbers reflect that one is not autonomously regulated for change whereas positive numbers reflect one is autonomously regulated to be active. In this study the Cronbachs Alpha was *α* = .88.

*Planning* was measured by eight items adapted to adolescents [27]. Example items worded “I have made a detailed plan regarding when and where to engage in regular moderate or vigorous physical activity” or “I have made a detailed plan regarding to what to do when running into bad weather or lack of sport resources”. All the items were scored on a four-point Likert scale ranging from (1) completely unable to (4) completely able. In this study the Cronbach,s Alpha was *α* = .93. 

*Physical activity behavior* in a usual week was measured using the 7-day PA recall questionnaire (IPAQ) adapted for Chinese adolescents [28]. Physical activity frequency, duration, and intensity were assessed; responses for frequency and duration were then multiplied to obtain an index of total physical activity per week. In this study test-retest reliability for IPAQ was *r* = .35, which is comparable to other studies conducted outside of China.

### 2.3. Data Analysis

Attrition analysis showed that the original sample at T1 (*N* = 693) did not differ from the follow-up sample (*N* = 534) in terms of sex, age, intention, autonomous regulation, and school or physical activity (all *P* > .05), showing that the 534 participants in the followup were a representative sample of the initial one. Pearson correlation analysis was conducted to examine the association between intention, autonomous regulation, planning, and physical activity. 

To test autonomous regulation's moderating effect on intention, planning, and posttest physical activity, a *mediation model* was specified with intention as the independent predicting variable, posttest physical activity as the dependent variable, and planning as a mediator between intention and physical activity. 

*Moderated mediation* was expressed by an interaction between intention and the index of autonomous regulation (intention × autonomous regulation) on behavior which affects the mediation process [29]. The analyses were based on procedures recommended by Preacher et al. [30] using the MODMEDC macro (Version 2.1; Model 2). To avoid multilinear influence, centered variables recommended by Aiken were used [31]. Missing data were imputed using the expectation maximization (EM) algorithm in SPSS [32]. A significance level of *P* < .05 was used throughout the analysis.

## 3. Results

Correlation analysis showed that all variables were significantly interrelated (see Table 1). Autonomous regulation proved discriminant validity with all other variables (*r* < .25), providing confirmation to include all variables in the subsequent analysis.

In the *mediator model*, T2 physical activity was significantly predicted by T1 intention, *β* = .25, *P* < .05. When T1 physical activity was included, intention accounted for 6% of the variance of physical activity change at T2. After T2 planning were included into the regression equation, T1 intention was not a significant predictor for T2 physical activity change anymore (*β* = .05, *P* = .22). However, baseline physical activity (*β* = .23, *P* < .05) and T2 planning (*β* = .35, *P* < .05) acted as significant predictors of follow-up physical activity. Thus, planning fully mediated the path from intention to behavior change. 

**Figure 1:**
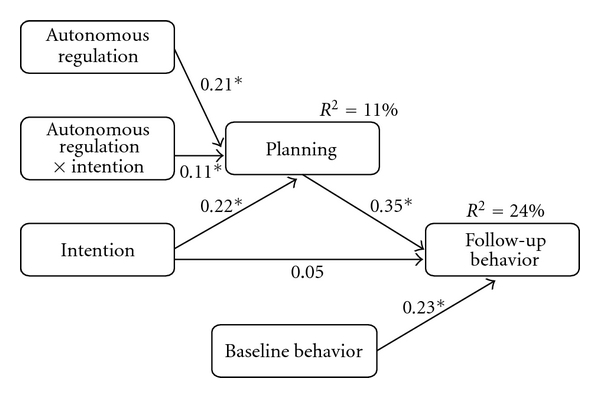
Moderated mediation model: planning as mediator between intention and physical activity behavior, and autonomous regulation moderating this mediation (results from the analyses using the MODMEDC macro Version 2.1; Model 2; cf. [28]; with centered variables). *Note*. *N* = 534, *P < .05, autonomous regulation, intention, and baseline behavior (physical activity) measured at baseline, planning and follow-up behavior (physical activity) four weeks later. Physical activity is the index of frequency × duration.

In the *moderated mediation model*, the moderator (T1 autonomous regulation) and the interaction variable (intention × autonomous regulation) were conjointly included into the regression equation. T1 intention significantly predicted T2 planning (*β* = .22, *P* < .05), together with autonomous regulation (*β* = .21, *P* < .05), and the interaction of intention and autonomous regulation (*β* = .11, *P* < .05). In total, 11% of the variance of T2 planning was explained. Baseline physical activity, T1 intention and T2 planning jointly accounted for 24% of the variance of T2 physical activity change (Figure 1). 

**Figure 2:**
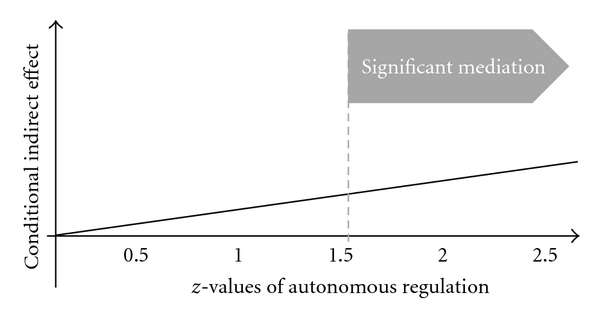
Indirect effect of intentions on behavior by mediation of planning, and the mediation moderated by autonomous regulation.

The significant interaction effect supported the assumption of a moderated mediation: planning mediated the intention-behavior relation, and this mediation was moderated by autonomous regulation. Follow-up analysis tested how high intention needed to be. Students required a mean value of at least 1.5 on the autonomous regulation scale to translate their intention into behavior via planning (*P* < .05) (Figure 2). 

Thus, the mediation effect of planning appeared to be conditional upon the value of autonomous regulation index. Only if autonomous regulation was 1.5 or higher, planning mediated significantly between intention and subsequent behavior.

## 4. Discussion

This study aimed at shedding more light on the mechanisms underlying physical activity change processes in adolescents. The mediating effect of planning as well as the moderating effect of autonomous regulation was confirmed: planning mediated the relation between intention and behavior, whereas the mediating effect was moderated by one's levels of autonomous regulation index. Those who perceived higher levels of autonomous regulation were more likely to translate their intentions into behavior change. This is consistent with many previous studies, for example with findings by Beauchamp et al. [6] that autonomous regulation is associated with higher levels of regular physical activity intention and self-efficacy. 

However, some limitations need to be mentioned. The study's four-week follow-up period is rather short. Furthermore, the study included only self-reported questionnaire measurements. Although this is a typically used procedure to measure physical activity among large samples [6], reporting bias might have occurred. In future studies, objective parameters of physical activity (e.g., pedometers, heart rate monitor etc.) can test the results' reliability. Above that, some of the measures were single items only. Also, prospect research need to test the revealed findings in experimental designs. 

Despite those limitations, some implications can be drawn from this study. Firstly, the moderated mediation model extended the understanding of factors associated with adolescent physical activity promotion: the effectiveness of planning mediation between intention and behavior change has been identified in some previous studies [15, 33]. However, planning was found to benefit not all participants equally [23, 24]. In this study the moderated mediation model reveals that planning intervention might be more effective among those adolescents with high autonomous regulation than among those with low autonomous regulation, which is important for health behavior educators who should take adolescents' current motivation status into consideration. As previous studies have mainly shown the importance of high intentions [23, 34] or high autonomous regulation [6], this study revealed the significant interaction of the two, that is, intentions and autonomous regulation. Autonomous regulation in turn offers opportunities for intervention, especially when teaching adolescents [6]. 

For health behavior promotion among adolescents, interventions matched to the characteristics of the adolescence might be more effective. If autonomous regulation is high, planning should be trained. However, if autonomous regulation is rather low, strategies to increase autonomous regulation are needed first. Such strategies are, for example to provide a training in transformational leadership to the teachers of students. This was done successfully by Beauchamp and colleagues [6].

Adolescence is a key phase for autonomy developing. Adolescents participate in physical activity, rather motivated by pleasure seeking and enjoyment (autonomous regulation) than by means of health gains and prevention of diseases [35, 36]. In planning intervention for adolescents, not only planning should be trained. Adolescents should also get help to increasing their autonomous regulation. This might be achieved by providing more choices, increase enjoyment experience and activity competence. With that their autonomous regulation might improve their long-term behavior adoption and maintenance, and accordingly their health. Thus, this might be an effective approach in preventive medicine. These findings are important because they add to the current knowledge of age-specific health promotion. Not only cognitive-rational factors are important to consider. But also affective factors such as autonomous regulation are crucial in adolescents. Hopefully, this opens avenues for effective prevention strategies across the life-span.

## Figures and Tables

**Table 1:** Means and standard deviations of as well as correlation between intention, action planning, autonomous regulation, and physical activity (PA) at two points in time.

	M	SD	1	2	3	4
(1) T1 intention	0.75	1.51	1			
(2) autonomous regulation	7.42	4.46	.28*	1		
(3) T1 PA	8.31	4.41	.43*	.27*	1	
(4) T2 Plan	2.78	0.72	.29*	.28*	.43*	1
(5) T2 PA	8.18	4.24	.25*	.35*	.13*	.19*

*P < .05, PA is the index of PA frequency × duration; T1, baseline; T2, follow-up four weeks after baseline.
